# Luxation of the Metacarpo-pharyngeal Articulation in a Horse

**Published:** 1897-07

**Authors:** W. L. Williams


### LUXATION OF THE METACARPO-PHARYNGEAL ARTICULATION IN A HORSE.

By W. L. Williams, V.S.

The comparative rarity of luxation without fracture in horses renders the following case of interest:

The patient was a common, medium-sized animal, used for wholesale grocery delivery, and while being driven at a trot over a railway crossing caught her left forefoot between the railroad rail and the crossing board, causing her to fall heavily forward on the imprisoned member.

The foot was pried loose with a crowbar. The animal got up, when the driver noticed that the fetlock-joint was out, but was readily replaced by him, and I was notified by telephone that the patient was on its way to our infirmary with its leg out of joint—" walking." On arrival at the infirmary the animal was walking comfortably without noticeable limping, no swelling or other injury about the joint being visible.

Picking up the leg and flexing it, the phalanges readily turned outward at a right angle to the metacarpus, and was as readily replaced. In other directions there appeared to be no abnormal mobility. The luxation and replacement caused no apparent pain or discomfort to the animal. She was walked home—a few blocks away—and the affected member placed in a firm plaster-of-Paris bandage reaching near to the carpus, which was allowed to remain for a week, the case going on without incident, and at the end of that time the bandage was removed and the animal returned to her accustomed work.

